# Effect of PbO-nanoparticles on dimethyl polysiloxane for use in radiation shielding applications

**DOI:** 10.1038/s41598-022-20103-z

**Published:** 2022-09-20

**Authors:** Ahmed M. El-Khatib, Mahmoud I. Abbas, Sabbah I. Hammoury, Mona M. Gouda, Kareman Zard, Mohamed. Elsafi

**Affiliations:** 1 Physics Department, Faculty of Science, Alexandria University, Alexandria, 21511 Egypt; 2 Head of Medical Physics and Radiotherapy Department, Alexandria Ayadi Almostakbal Oncology Hospital, Alexandria, Egypt

**Keywords:** Materials science, Physics

## Abstract

In this work, morphological and attenuation parameters of gamma ray protection were studied. Dimethyl polysiloxane (Silicon Rubber) Mixed with micro-size and nano-size lead oxide particles at different weight percentage were prepared. The morphological structure of PbO/SR composites was investigated by SEM test, according to SEM images the nano PbO particles are more uniform micro PbO particles. The radiation attenuation test was carried out using 3ʺ × 3ʺ NaI (TI) detector for (Am-241), (Cs-137), (Co-60), (Ba-133), and (Eu-152). The effect on attenuation property of SR-PbO shown that the increase of PbO filler significantly increases the linear attenuation coefficient and improve the other radiation protection parameters especially at low gamma energy. It's found that a significant agreement between the experimental result and theoretical result from Xcom program. In this study it's found matrix filled with nano-PbO have higher gamma shielding ability compared to micro-PbO matrix at the same filler concentration. It can say that SR-nano PbO has a higher radiation protection than SR-micro PbO compositions.

## Introduction

Gamma rays have a short wavelength which makes the gamma rays good energy and more power than other electromagnetic waves. Gamma rays are used in the treatment of cancer. Radiation oncology or radiation therapy using gamma rays to control or kill malignant tumor cells in patients. However, the use of gamma rays not only damage, cancer cells, but also healthy cells is killed through the process which called organs at risk. To provide radiation protection to prevent reactions with healthy organs in the patient and to provide protection to radiation worker^1,2^.

The radiation shield is required, which is made of a material with a high atomic number such as lead (Pb), Cadmium (Cd), Tin (Sn), and bismuth (Bi). However, the use of this material has a disadvantage one of which has no flexibility where it cannot protect the patient's body when it will receive radiotherapy. In addition, it’s not provided radiation protection garments with lightweight, comfortable and easy to use^3,4^.

Polymers are large molecules formed by the joining many of monomers together. The most advantage of polymer materials is that they have low density compared to common shielding materials and increase corrosion resistance. Silicone rubbers are one of the synthetic polymers derivatives from polydimethylsiloxane which has good elasticity properties, high strength, size and thermal stability. In addition to all these advantages their lightness gives silicon rubber superiority in many areas. For these reasons, polymer used as matrix materials to get flexible radiation protective materials where high atomic number(Z) material as lead, Cadmium, Tin, and bismuth powders are used for additives. In recent years, nanomaterials are commonly used to enhance the radiation attenuation properties of shielding material which matrix polymers such as epoxy which is more convenient rigid structures, whereas silicon rubber suitable for flexible wearable materials^5,6^.

A lot of the materials used for gamma-irradiation shielding to contain elements such as lead, titanium, bismuth, tin, concrete, cadmium, paraffin, and polymers. PbO selected as a contributing material to perform better radiation attenuation. The addition of PbO nanofillers in the silicon rubber matrix will stepwise attenuates gamma ray irradiation. Because of the low electron density of nanomaterial compared to bulk materials, high-Z nanoparticles added nanomaterial will produce comparatively low bremsstrahlung, optimize mechanical, thermal and electrical properties of the silicon matrix compared to the bulk material. Silicon rubber matrix with nanomaterials can improved gamma ray attenuation characteristics of nanocomposites since nanomaterials are more uniform and have a less clout in the composite and so it can enhance the protective ability of material^7–9^.

In this study the lead oxide embedded in the silicon rubber material. The matrix was produced and tested against Am^241^, Cs^137^, Co^60^, Ba^133^, and Eu^152^ gamma source. The characteristic properties of composites were tested by SEM scan and checked the size of lead oxide particle for micro and nano particle using TEM imaging. In this work we studied many shielding parameters of the material and protection performance in composites and compare flexible and lighter silicon rubber products between micro and nano filler and comparing radiation protection properties of materials with same weight fraction.

## Materials and methods

### Samples

Silicon rubber was used as a polymer where the original state is liquid. By using catalyzed cross-linking reactions silicon are converted to elastomeric solid structure about 2 wt% stiffener was added to polymer liquid. The homogeneous mixture molded in cylinder which have 3 cm diameter with different thickness, after waiting 24 h the mixture become elastomeric solid material^10,11^.

**Figure 1:**
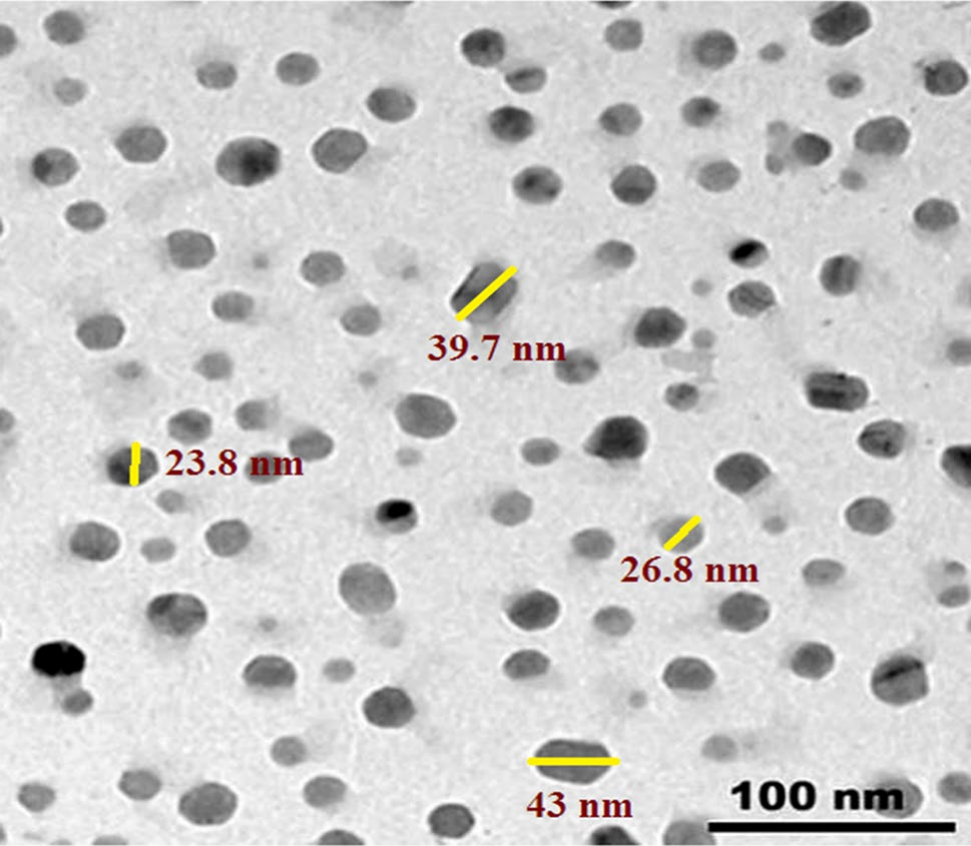
TEM image of nano-lead oxide particles.

In this work. Micro and nano size lead oxide particles were used as a filler additive. Lead oxide in micro size was purchased from Elgomhira Co, (Egypt) and nano-lead oxide was supplied by Nanotech Co, (Egypt). The Field Emission Transmission Electron microscope (FE-TEM) [JEM-2100F, JEOL, Japan] at 200 kv used to estimate particle size. The measured particles were prepared by distributing the material in ethanol by ultrasonic vibration on a gold grid. The mean particle size was obtained by intermediated a number of measured diameters in the detected image of the sample. Figure 1 display the image of the lead oxide nanoparticle which have range size from 50.7 to 19.5 nm with standard deviation 14.36 while the micro particle have average size 22 ± 5 μm^12–14^.

In addition, lead oxide concentrates powder were added to silicon rubber then adding stiffeners in solidification process. The preparation of four different concentrations of micro SR/PbO composites, two samples of nano PbO/SR were imbedded in silicon rubber as shown in Table 1. The sample was named according to the lead and the ratio in the sample. By measuring mass and calculate volume with Arcimedes’ principle The density of silicon rubber sheets was calculated using the equation^15^.

**Table 1:** Code and density of samples.

Sample code	Silicon rubber (wt%)	Lead oxide (wt%)		Density (gm/cm^3^)
		Micro	Nano	
SR-0	100	–	–	1.181 ± 0.081
SRM-5	95	5	–	1.304 ± 0.009
SRM-10	90	10	–	1.386 ± 0.015
SRM-20	80	20	–	1.414 ± 0.022
SRM-50	50	50	–	2.038 ± 0.005
SRN-20	80	–	20	1.460 ± 0.009
SRN-50	50	–	50	2.086 ± 0.074

1 $$\rho =m/v$$

where, $\rho$ is the sheet density (g/cm^3^), m is mass of sheet (g) and V is sheet volume (cm^3^).

### Morphological images

In the work, the image was used to observe the distribution of micro and nano PbO particle and cross-section morphologies of PbO/SR composites. A Scanning Electron Microscope (SEM) [JSM-6010LV, JEOL] is based on scanning the sample where the electron beam focused over a sample surface to create images. The electron beam interacts with the sample producing various signals that used to obtain information about the cross section topography and composition^16,17^.

### Mechanical measurements

The mechanical properties were tested to explain the effect of stress–strain behavior of free and filled silicon rubber samples by study the relationship between stress and strain. The ultimate force and break distance for the SR-PbO matrix were determined using Generic Compression^18^.

### Instrumentation

**Figure 2:**
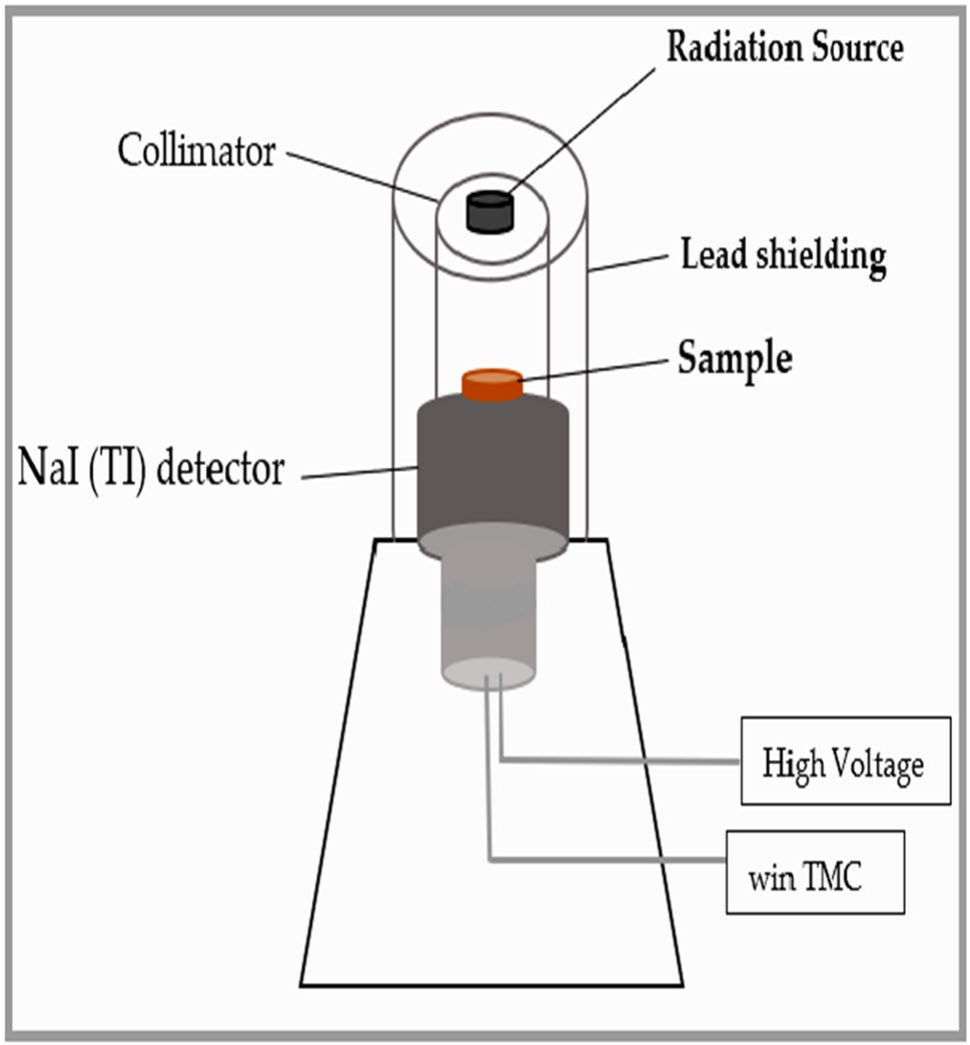
The arrangement of the radiating system.

**Figure 3:**
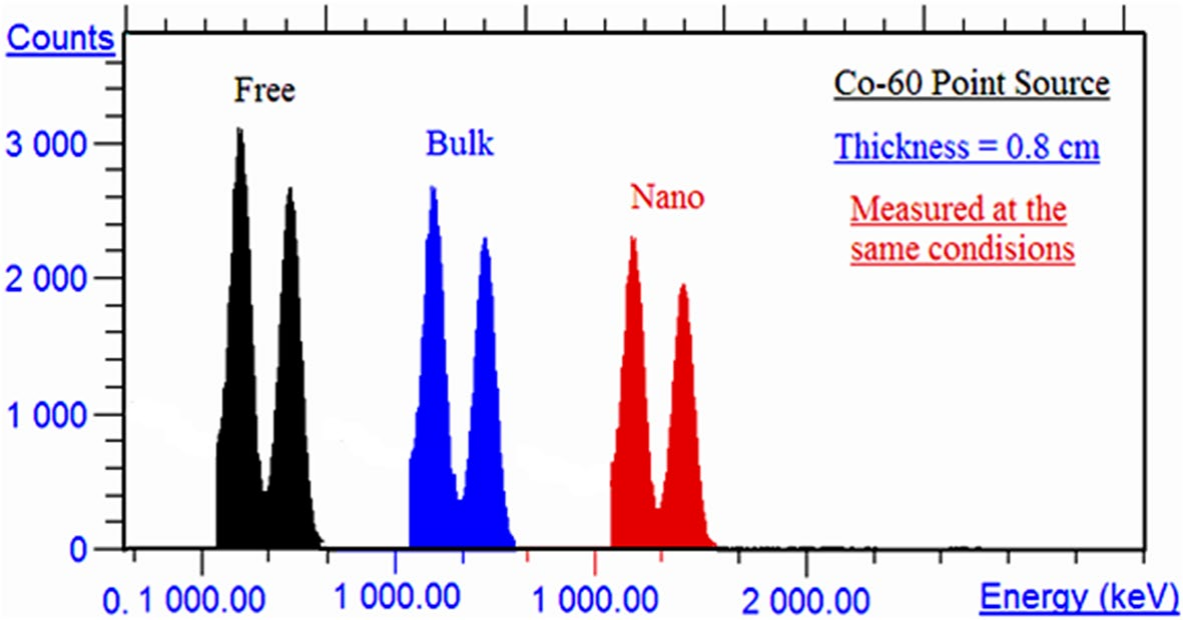
Spectrum of counts for Co-60 Source in case without absorber (Black color), with SRM-20 absorber sample (Blue color) and with SRM-20 absorber sample (Red color).

The design and arrangement of the radiating system “the detector, radioactive source and attenuation material sample” are explained in Fig. 2. The irradiations were carried out using the narrow beam technique of gamma ray spectroscopy. Using NaI (TI) cylindrical detector of dimension 3ʺ × 3ʺ which was surrounded and cylindrical by a lead shielding of 40 mm thickness and 135 mm height to minimize the background radiation. The distance between a radioactive source and the surface of the detector was 112 mm for many reasons as obtain very narrow beam to get an approximately parallel beam, ignore the effect of detector dead time and reduce the impact of coincidence summing. The attenuation composites were placed on a holder between the radioactive source and detector for a sufficient exposure time until the error is less than1%. The characteristic peak in each spectrum was amplified, then analyzed by win TMC software. The linear attenuation coefficient μ is one of the main parameters for estimating the impact interaction of gamma radiation with shielding material which calculated according to Beer–Lambert low^19–23^.

2 $$I=I_{o} {e}^{-\mu x}$$

where ${I}_{n}$, initial intensity of gamma ray, *I* transmitted intensity gamma ray, $x$, thickness of the sample, where the intensities were calculated experimentally by calculate the net count rate at each energy in with and without samples using WinTMC software as shown in Fig. 3 as an example of SR-20PbO bulk and nano. The adequation of the interested polymer can be tested as a radiation shielding material by calculating the half value layer (HVL) and tenth value layer (TVL) where they are important factors in designing a convenient radiation protecting material and its thickness. The half value layer is the material thickness enough to reduce the gamma ray intensity to 50% of its initial intensity

3 $$HVL=\frac{LN (2)}{\mu }$$

The tenth value layer is the material thickness enough to reduce the gamma ray intensity to 10% of initial intensity

4 $$TVL=\frac{LN (10)}{\mu }$$

The mean free path (MFP) is defined as the average distance between two successive collisions between photon and the polymer matrix

5 $$MFP=\frac{1}{\mu }$$

And calculate the relative deviation for experimental linear attenuation coefficient of micro filler compared to the XCOM result (Dev1) and between micro experimental data and nano experimental data (Dev2).

6 $$Dev1=\frac{XCOM - EXP}{EXP}\mathrm{X }100$$

7 $$Dev2= \frac{Nano - Micro}{Micro} \mathrm{X}100$$

The radiation protection efficiency (RPE) is an important parameter for estimating the efficacy of shielding materials

8 $$RPE\%=[1-\frac{I}{I_{o}}]\times 100$$

The linear attenuation coefficient and other parameter values of the SR/PbO samples were measured experimentally in the Radiation Physics Laboratory, Faculty of Science, Alexandria University. The radioactive sources [^241^Am, ^133^Ba, ^60^Co, ^137^Cs and ^152^Eu] used to generate gamma rays purchased from Physikalisch-Technische Bundesanstalt PTB in Braunschweig and Berlin in range from 59.53 to 1408.01 keV^24–28^.

### Result and discussion

## Scanning electron microscope

**Figure 4:**
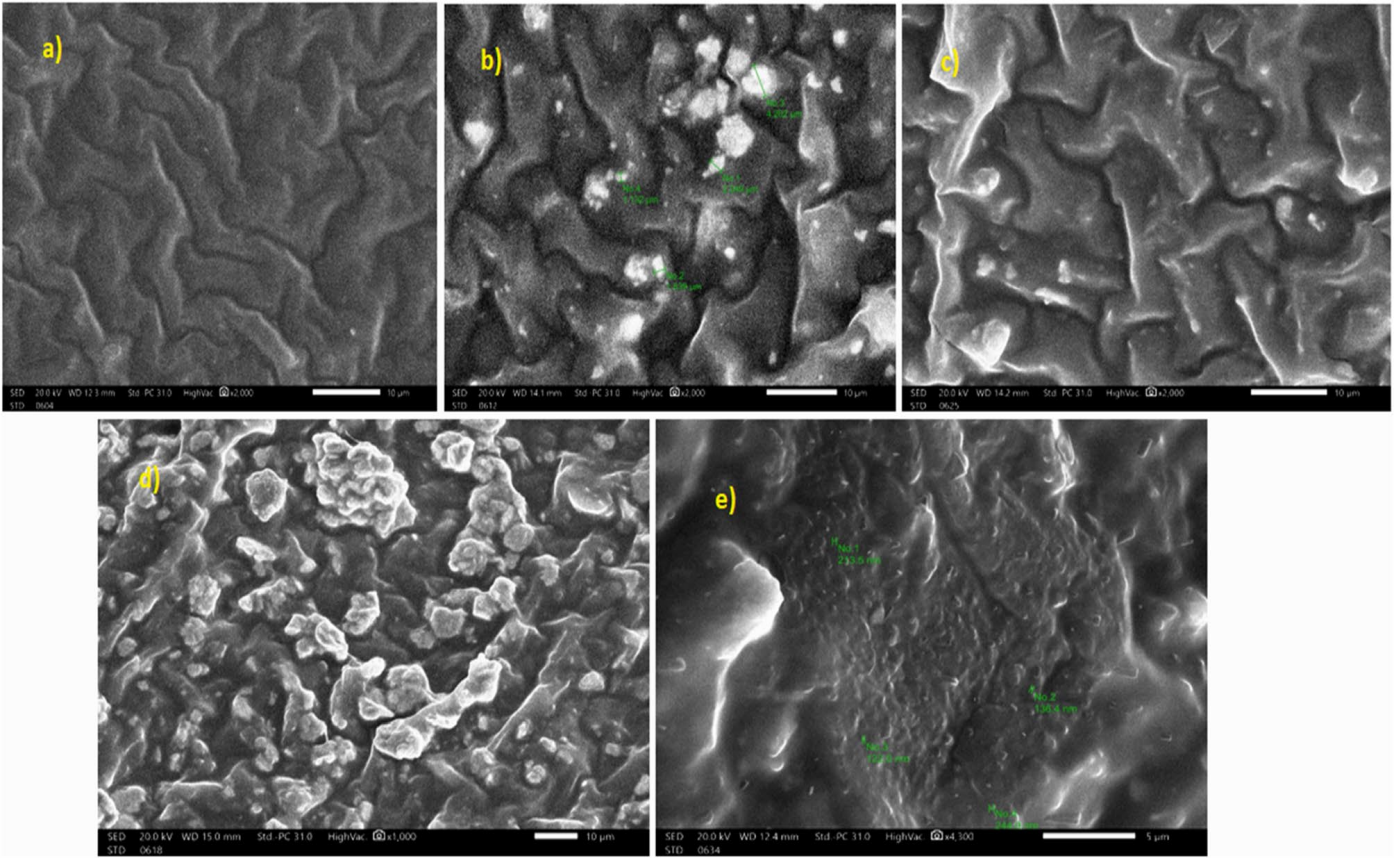
SEM image of (**a**) pure dimethyl polysiloxane (silicon rubber), (**b**) 20 wt% micro PbO sample, (**c**) 20 wt% nano PbO sample, (**d**) 50 wt% micro PbO micro, (**e**) 50 wt% nano PbO sample.

The SEM image of the pure silicone rubber, micro PbO, and nano PbO. Silicon rubber (dimethyl polysiloxane) filled with 20 wt% micro PbO, 20 wt% nano PbO, 50 wt% micro PbO, and 50 wt% nano PbO are displayed in Fig. 4, which used to explain the effect of micro and nano lead oxide particle on silicon rubber. Figure 4a show smooth and definite variation than SR/PbO composites Fig. 4b–e while the filler face to form conglomerates at higher mass. As increase lead oxide content, the conglomerates will be visible and more sophisticated. Figure 4b,c and d,e SEM images of micro and nano SR/PbO matrix sample with the same weight percentage of filler are compared. It represents in case of 20 wt% and 50 wt% nano filler sample, PbO nanoparticle is distributed homogeneous, which increase interpenetration between SR and PbO nano particle. It provides cobwebbed structure for protection. In case of 20 wt% and 50 wt% micro SR/PbO composites, the micro PbO particle is not mixed well with SR matrix which led to decreased interpenetration. From the SEM images, the dispersion of nano particle more homogenous and uniform than micro particle these led to more protection performance in nano sample.

### Mechanical measurements result

**Figure 5:**
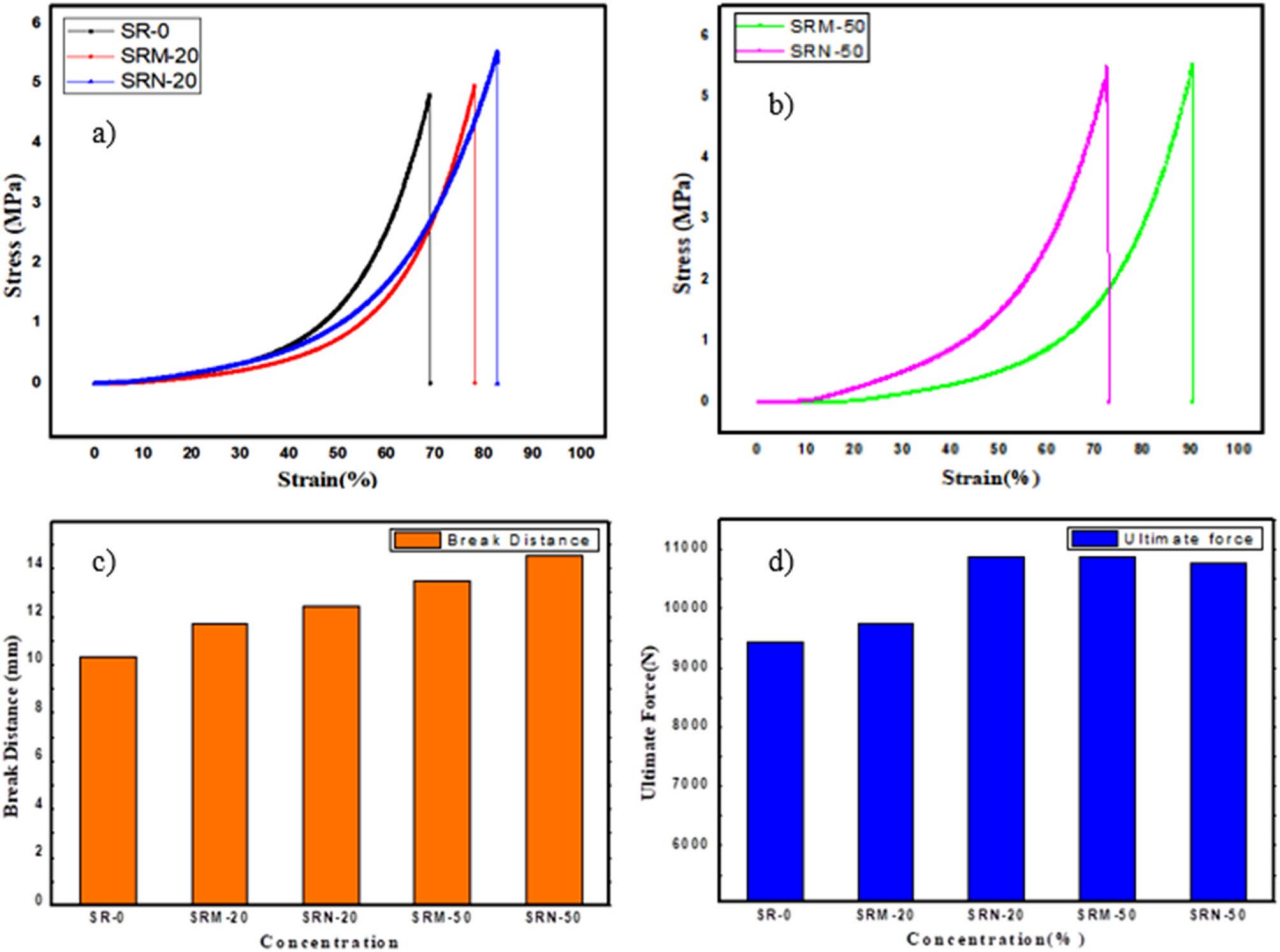
The mechanical properties of free silicon rubber and with different concentration of micro and nano lead oxide (**a**) Stress-Strain curve for SR-0 and both SR-20, (**b**) Stress-Strain curve for SR-50, (**c**) break distance as a function of PbO concentration, (**d**) Ultimate force as a function of PbO concentration.

The mechanical properties of the prepared materials at room temperature were shown in Fig. 5. These graphs explain the variability of the stress—strain curve, ultimate force, and the break distance with different concentrations of micro and nano lead oxide as fillers. Figure 5a and Fig. 5b showed the results of stress vs. strain relationship and was observed from the dynamic compressive test of free silicone rubber relatively poor compared to filled samples, where nano composites are better stress effect than micro composites in the same concentration. Figure 5c and d showed that when the filler increase, it will lead to a significant increase in break distance and ultimate force.The results showed that the addition of nano-lead oxide gives an increase in break distance and ultimate force than micro-lead oxide with the same percentage. Low mechanical properties at 50 wt% of filler concentration is due to agglomerates, interfacial, and accumulation of filler material in different rubber layers, it’s meant that 50% represents the saturation point where hardness was increased with lead oxide concentration increase.

### Gamma ray attenuation results

The linear attenuation coefficient LAC is the main parameter used in this study that using to evaluate the efficiency of the radiation protection material. Table 2 explain the value of experimental LAC, theoretical LAC get from Xcom software, and measured density of 0wt% PbO silicon rubber, micro SR/PbO, nano SR/PbO matrix. The relative deviation percentage (Dev1) between LAC of Xcom value and experimental result and (Dev2) between LAC of nano and micro matrix is listed in Table 2. It’s observed that the LAC of micro SR/PbO has significal agreed with the Xcom data. The experimental result shows that LAC value increases significally with increasing percentage of both micro and nano PbO in the sample and decrease sharply an energy increase in the range from 59.53 to 1408 keV. Because of the increase of the density of the sample, that means the high Z lead filler elaborate the radiation protection properties. It’s can be explained due to the transition of gamma ray through the material has three main interactions are photoelectric effect, Compton scattering, and pair production where at energy less than 125 keV, the interaction probability for photoelectric effect is scientifically high and photons tend to absorbed by the photoelectric effect according to Z^3^/E^3^. So that as the concentration of PbO in the matrix increase, the LAC increase where lead is one of the higher shielding elements on the other side, where photoelectric effect probability is indirectly proportional to E^3^, LAC decrease sharply with increase the energy of the photon.

**Table 2:** Measured density, linear attenuation coefficients, relative deviation, and theoretical Xcom value for pure SR, micro SR/ PbO, and nano PbO/SR composites.

Sample	Energy (keV)	LAC, cm^−1^					Density (g cm^−3^)	
		Micro			Nano		Micro	Nano
		XCOM	EXP	Dev1	EXP	Dev2		
SR-0	59.51	0.2652	0.2628	0.92			1.181	
	80.99	0.2175	0.2096	3.76				
	121.78	0.1848	0.1766	4.62				
	244.69	0.1461	0.1430	2.18				
	356.01	0.1076	0.1045	2.94				
	661.66	0.0984	0.0976	0.77				
	778.91	0.0921	0.0920	0.12				
	964.13	0.0826	0.0793	4.16				
	1173.3	0.0749	0.0739	1.48				
	1332.2	0.0702	0.0688	2.03				
	1408.1	0.0683	0.0665	2.71				
SRM-5	59.51	0.5738	0.5686	0.91			1.304	
	80.99	0.3615	0.3539	2.16				
	121.78	0.4079	0.3894	4.76				
	244.69	0.1915	0.1873	2.23				
	356.01	0.1507	0.1448	4.08				
	661.66	0.1100	0.1084	1.46				
	778.91	0.1024	0.1032	− 0.83				
	964.13	0.0912	0.0909	0.39				
	1173.3	0.0825	0.0821	0.50				
	1332.2	0.0772	0.0740	4.39				
	1408.1	0.0751	0.0728	3.16				
SRM-10	59.51	0.9084	0.8789	3.35			1.386	
	80.99	0.5132	0.4990	2.84				
	121.78	0.6503	0.6304	3.16				
	244.69	0.2357	0.2197	7.31				
	356.01	0.1711	0.1660	3.09				
	661.66	0.1183	0.1186	− 0.29				
	778.91	0.1097	0.1075	2.02				
	964.13	0.0970	0.0958	1.20				
	1173.3	0.0874	0.0860	1.68				
	1332.2	0.0818	0.0788	3.73				
	1408.1	0.0794	0.0764	4.01				
SRM-20	59.51	1.5360	1.5002	2.39	1.7923	16.30	1.414	1.460
	80.99	0.7865	0.7598	3.52	0.7796	15.37		
	121.78	1.1056	1.0994	0.56	1.1644	14.42		
	244.69	0.3058	0.2992	2.19	0.3280	13.03		
	356.01	0.1969	0.1859	5.90	0.2118	12.21		
	661.66	0.1235	0.1226	0.80	0.1364	10.12		
	778.91	0.1131	0.1148	− 1.49	0.1272	9.73		
	964.13	0.0990	0.0969	2.17	0.1057	8.37		
	1173.3	0.0886	0.0898	− 1.27	0.0969	7.36		
	1332.2	0.0827	0.0821	0.75	0.0879	6.67		
	1408.1	0.0803	0.0785	2.22	0.0839	6.42		
SRM-50	59.51	4.7628	4.7111	1.10	5.8901	29.08	2.038	2.086
	80.99	2.2704	2.1922	3.56	2.8428	31.33		
	121.78	3.5054	3.3379	5.02	4.5154	30.51		
	244.69	0.7235	0.6915	4.62	0.9225	29.37		
	356.01	0.3803	0.3658	3.95	0.6383	28.59		
	661.66	0.1905	0.1924	− 0.99	0.2606	26.17		
	778.91	0.1692	0.1673	1.14	0.1979	25.58		
	964.13	0.1428	0.1377	3.72	0.1821	24.37		
	1173.3	0.1253	0.1247	0.49	0.1633	23.62		
	1332.2	0.1161	0.1164	− 0.29	0.1506	22.70		
	1408.1	0.1125	0.1100	2.34	0.1417	22.40		

**Figure 6:**
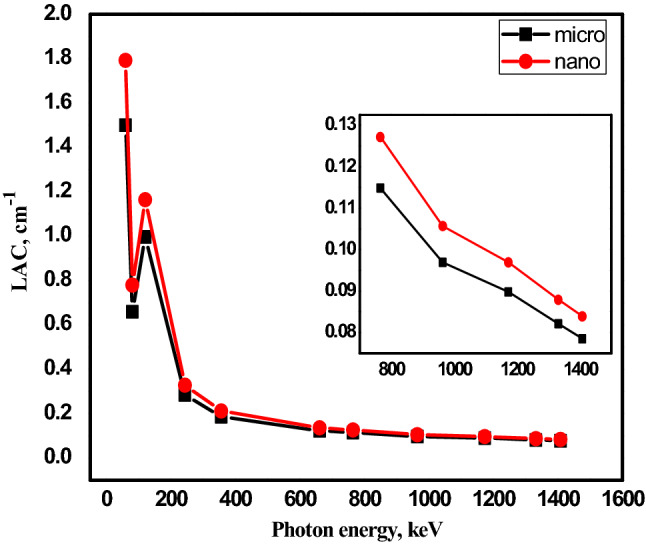
Comparison between LAC for nano- and micro-PbO for 20 wt% at different energy photon.

**Figure 7:**
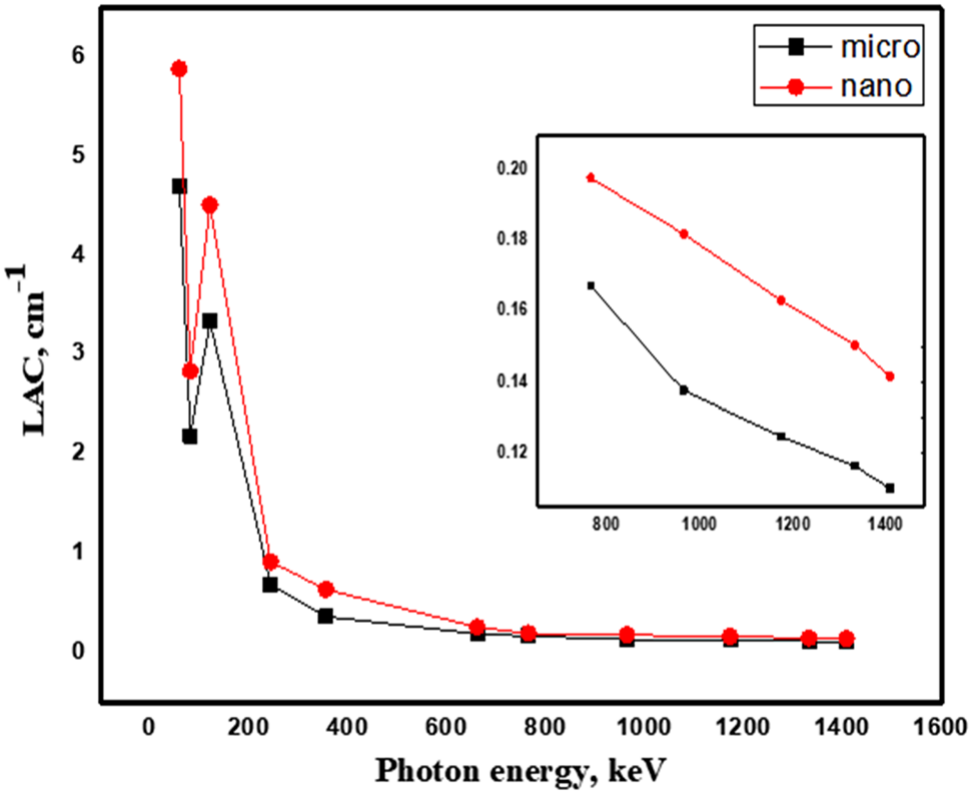
Comparison between LAC for nano- and micro-PbO for 50 wt% at different energy photon.

**Figure 8:**
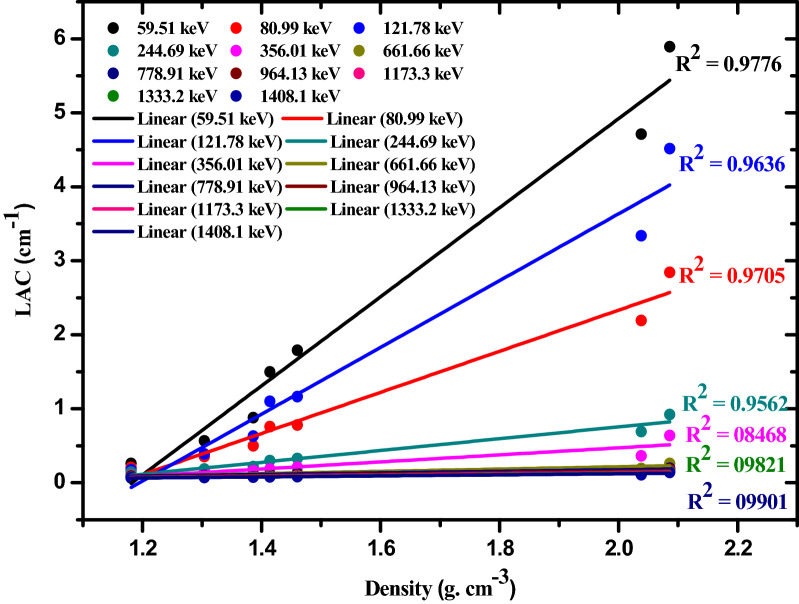
The relationship between LAC for nano- and micro-PbO and their densities at different energy photon.

Figures 6 and 7 depicts the variation of the linear attenuation coefficient between nano PbO/SR composites and micro PbO/SR composites with 20 wt% and 50 wt% as a function of gamma energy. It’s observed that, nano PbO/SR composites have higher LAC than micro PbO/SR for the same concentration of all the interested gamma-rays where particle size decrease from micro to nano, the particle distribution will be more uniform within polymer composites. So that the interaction probability between incident photon and nano-PbO particle will be increase compared with interaction between incident photon and micro-PbO particle. So that for the same PbO concentration and the same chemical structure, nano-PbO particle represents more attenuation protection than micro-PbO particle. Density is one of the the main physical parameter which characterize shielding material which effected by particle size, type, and weight fraction of reinforced material with polymer. Nano-composites densities are higher than micro-composites densities for the same weight fraction and as weight fraction of lead oxide increase densities increase. Figure 8 explains the relation between the linear attenuation coefficient and densities of samples at different energy where it’s clear that as density of samples increase the linear attenuation coefficient increase significally in low energy but it’s not significant at high energy. The linear correlation coefficients are (0.982, 0.985, 0.981, 0.977, 0.920, 0.951, 0.977, 0.945, 0.946, 0.951, 0.950) between densities and energies (60, 81, 121, 356, 662, 765, 964, 1173, 1332, 1408) keV respectively that mean there are strong relation between linear attenuation coefficient and density of composites.

**Figure 9:**
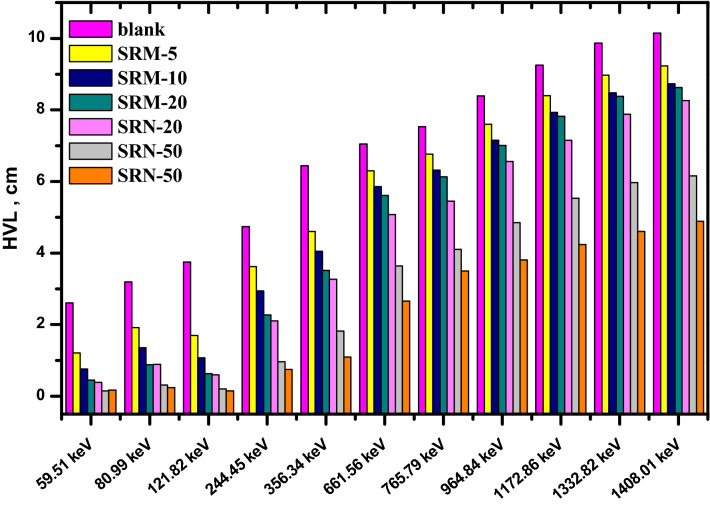
HVL of pure silicon rubber, micro, and nano PbO at different energy.

Where, half value layer is one of the main parameters calculated in designing a suitable radiation shielding. The half value layer of pure silicone rubber, micro-PbO, and nano-PbO at different gamma energies are calculated and represent in Fig. 9. According to table result, the pure silicon rubber has much higher HVL than the investigated composites and as filler concentration increase the shielding properties increase and the HVL value decrease. As photon energy increase, the HVL value increase, which led to more thickness of the shielding material.

**Figure 10:**
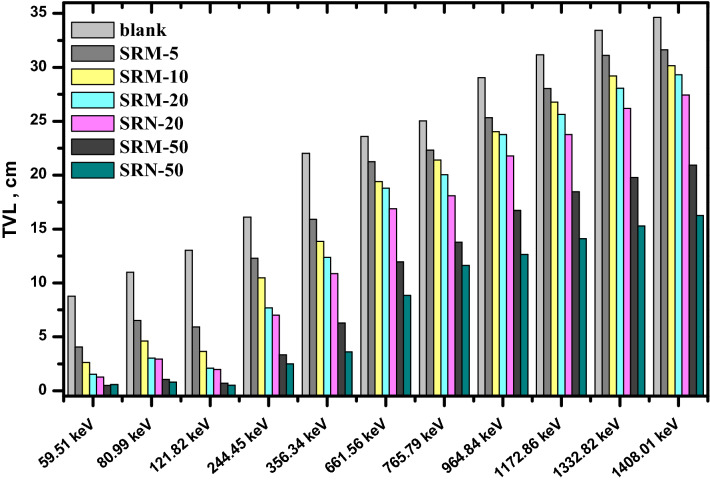
TVL of pure silicon rubber, micro, and nano PbO at different energy.

**Figure 11:**
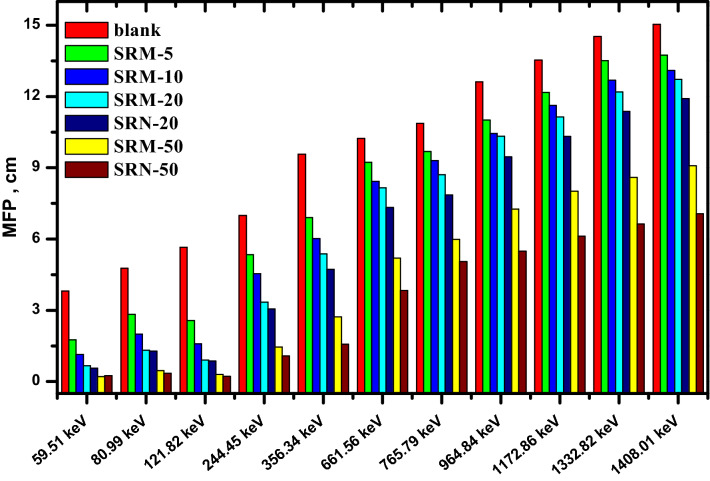
Mean free path value at different concentration and at different energy.

**Figure 12:**
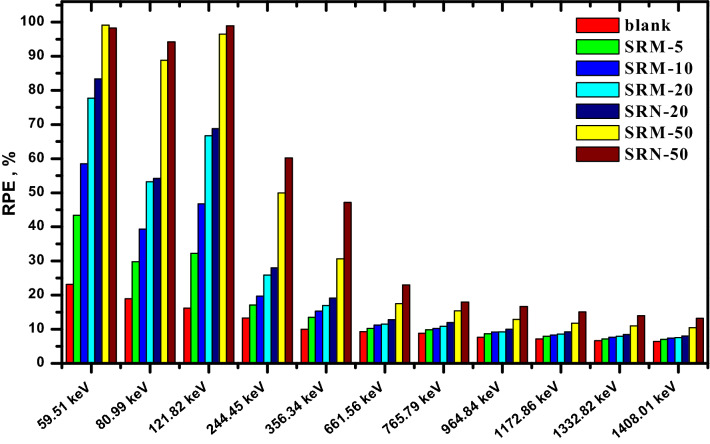
RPE of pure silicon rubber, micro, and nano PbO at different energy at thickness 1 cm.

It’s explained that, the composites which have the same filler concentration, the nano-PbO/SR composites have HVL lower than the micro-PbO/SR composites at all investigated energies. Similarly, the tenth value layer and mean free path were estimated in Figs. 10 and 11, respectively. The TVL and MFP values explain the nano composites have higher protecting performance than micro composites. In Fig. 12, the result of radiation protection efficiency, which measure the efficiency of shielding material at thickness 1 cm.

**Figure 13:**
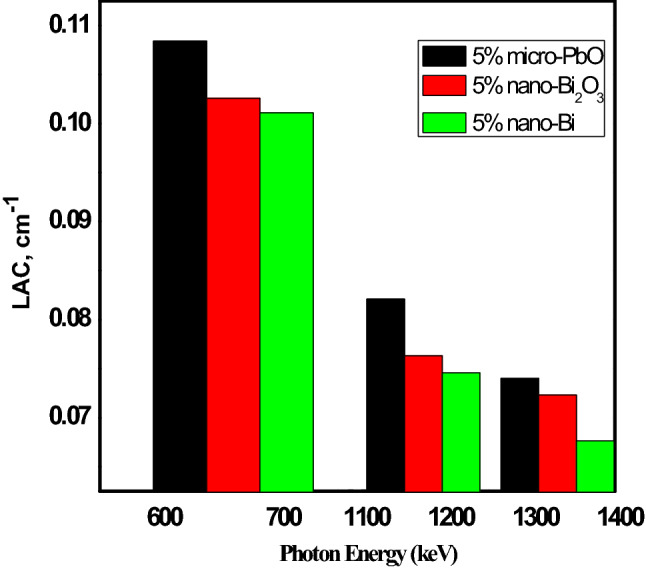
Comparison the LAC between our result and 5 wt% of micro-PbO/SR, nano-Bi_2_O_3_/unsaturated polyester, and nano-Bi/unsaturated polyester at 661, 1172 and 1332 keV.

**Figure 14:**
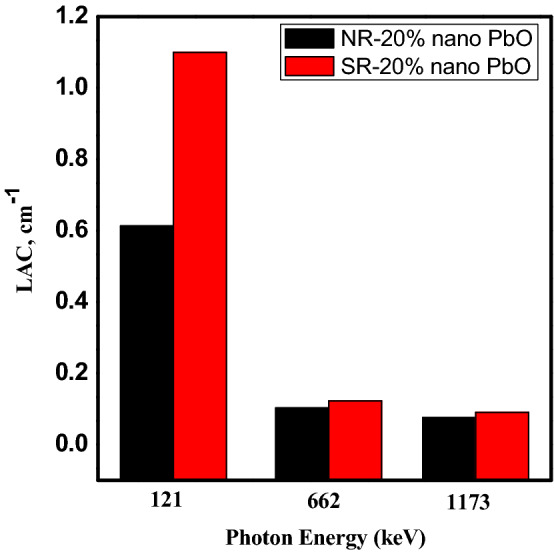
Comparison the LAC between our result for 20 wt% of nano-PbO/Silicon Rubber and 20 wt% of nano-PbO/Natural RUBBER at 121.8, 661 and 1173.2 keV.

Abdul et al.^15^, studied the effect of adding Bi and Bi2O3 nanoparticles to silicon rubbers to investigate its efficiency as a gamma ray shielding. In Fig. 13 compared the linear attenuation coefficient values at 662 keV, 1172 keV, and 1332 keV between result shown in reference^15^ for unsaturated polyester witch reinforcement with 5 wt% Bi and 5 wt% Bi_2_O_3_ nanoparticles and silicon rubber reinforced with micro-lead oxide at 5 wt% concentration. The figure showed that micro-lead oxide as filler material for polymer has a higher linear attenuation coefficient for all investigated energy at the same weight fraction. In Fig. 14 compared the linear attenuation coefficient values at 121, 662, and 1173 keV between result shown in El-khatib et al.^7^ where natural rubber and silicone rubber filled with nano-lead as additive material at 20 wt% concentration. The figure Explain that silicon rubber has a higher linear attenuation coefficient compared to natural rubber when they have filled with nano-lead with the same weight fraction, where the densities were 1.22 and 1.460 g cm^−3^ for natural rubber and silicon rubber, respectively. It’clear that the shielding properties depend on the density of samples, where the LAC increases with increasing the density.

## Conclusion

In this study, micro Pb/SR and nano PbO/SR composites were prepared to study the effect of particle size and weight concentration of PbO particles against gamma ray by different radiation protection parameters. The morphological structure of PbO/SR composites was investigated by SEM test, according to SEM images the nano PbO particles are more uniform micro PbO particles. The experimental result was explained that size and weight percentage of the PbO affected the density of composites where as density increase linear attenuation coefficient increase gradually. The nano PbO composites have a higher linear attenuation coefficient compared to micro PbO composites, the half value layer and the tenth value layer in nano composites is lower than micro composites at the same weight concentration. we concluded that a new composites has been obtained that has a high radiation attenuation coefficient with specific features such as light weight and high flexibility, which can be used to protect some organs such as eyes, gonads and breasts during treatment or radiological diagnosis as it absorbs a large proportion of the incident rays.

## Data Availability

All data generated or analyzed during this study are included in this published article.
